# Association between the perivascular fat attenuation index and CCTA-defined anatomically significant left anterior descending coronary artery stenosis: relationship with serum inflammatory biomarkers

**DOI:** 10.3389/fcvm.2026.1823667

**Published:** 2026-10-19

**Authors:** Yiliya Ahongjiang, Alimujiang Abudukaiyoumu, Gulinuer Abulimiti, Hamisiguli Tuerxun, Aibibulajiang Mutailifu

**Affiliations:** 1 Department of Radiology, The First People’s Hospital of Kashi Prefecture, Kashi, China; 2 Centre for Translational Medicine, Nanjing Medical University Affiliated Kizilsu People’s Hospital, Artux, Kizilsu, China

**Keywords:** coronary artery stenosis, coronary inflammation imaging, inflammatory biomarkers, left anterior descending artery, non-invasive diagnosis, perivascular fat attenuation index

## Abstract

**Objective:**

To investigate whether lesion-specific perivascular fat attenuation index (FAI) is associated with CCTA-defined anatomically significant (≥50%) left anterior descending coronary artery (LAD) stenosis and to evaluate the diagnostic performance of a composite model integrating serum inflammatory biomarkers.

**Methods:**

We retrospectively analyzed 160 patients with suspected coronary artery disease (CAD) who underwent coronary computed tomography angiography (CCTA). Patients receiving anti-inflammatory medications were excluded. Lesion-specific perivascular FAI was measured around the LAD. The prespecified primary outcome was CCTA-defined anatomically significant LAD stenosis (≥50%), analysed as a binary variable. Multivariable logistic regression identified independent predictors, and the final model was internally evaluated with stratified 5-fold cross-validation, 10-fold cross-validation, and 1,000-bootstrap resampling. Model building was repeated within each resample. Calibration (slope, calibration-in-the-large, Brier score) and incremental discrimination (DeLong test) were assessed.

**Results:**

Patients with CCTA-defined anatomically significant stenosis (≥50%) had higher, less negative FAI values, higher hs-CRP and LDH levels, and a lower ApoA1/ApoB ratio than those with stenosis <50% (all *P* < 0.05). FAI correlated with stenosis both as a continuous percentage (*ρ* = 0.54, *P* < 0.001) and as a binary ≥50% variable (*ρ* = 0.64, *P* < 0.001), with less negative FAI values accompanying more severe stenosis. The final model identified FAI (OR = 1.055, 95% CI: 1.014–1.098), hs-CRP (OR = 1.063, 95% CI: 1.005–1.125), LDH (OR = 1.014, 95% CI: 1.005–1.023), and the ApoA1/ApoB ratio (OR = 0.209, 95% CI: 0.070–0.624) as independent predictors. The composite model achieved an AUC of 0.915 (95% CI: 0.865–0.965); internal validation yielded AUCs of 0.872 (5-fold), 0.881 (95% CI: 0.826–0.936; 10-fold), and 0.895 (95% CI: 0.848–0.942; bootstrap). On formal DeLong testing for correlated ROC curves, the composite model showed significantly better discrimination than hs-CRP alone (*P*  =  0.020) and than FAI alone (*P* < 0.001).

**Conclusions:**

Higher, less negative perivascular FAI values were associated with CCTA-defined anatomically significant LAD stenosis. A composite model integrating FAI, hs-CRP, LDH, and the ApoA1/ApoB ratio showed high apparent diagnostic accuracy and remained stable across multiple internal validation strategies. Because this was a single-centre retrospective study without external validation or outcome data, these findings should be regarded as preliminary and hypothesis-generating; the model is not ready for clinical use.

## 1 Introduction

Coronary artery disease (CAD) remains a leading cause of morbidity and mortality, and stenosis severity alone incompletely captures plaque biology and downstream risk. Coronary computed tomography angiography (CCTA) provides noninvasive assessment of coronary anatomy, while perivascular fat attenuation index (FAI) offers a complementary imaging biomarker of coronary inflammation. Because adipose attenuation values are negative, a higher FAI denotes a less negative CT attenuation value and biologically indicates a greater local inflammatory burden in perivascular adipose tissue (1–6).

Systemic inflammatory and metabolic biomarkers may further refine risk assessment. High-sensitivity C-reactive protein (hs-CRP) reflects low-grade inflammatory activation, lactate dehydrogenase (LDH) has been linked to adverse cardiovascular outcomes in some cohorts, although the direction and magnitude of this association remain controversial (7, 8), and the ApoA1/ApoB ratio reflects the balance between antiatherogenic and atherogenic lipoprotein activity (7, 9–15). Integrating these circulating markers with lesion-specific FAI may therefore provide a more biologically informed assessment than either anatomic stenosis or serum biomarkers alone.

Regional heterogeneity in perivascular adipose tissue provides an additional rationale for vessel-specific analysis. Prior work has shown that FAI varies across coronary territories, including differences between the right coronary artery and the LAD (16). To reduce anatomical confounding, this study focused on LAD lesions and examined whether lesion-specific FAI, alone and combined with serum inflammatory biomarkers, is associated with CCTA-defined anatomically significant stenosis.

The primary objective of this study was therefore to determine whether lesion-specific perivascular FAI is associated with the *presence* of CCTA-defined anatomically significant (≥50%) LAD stenosis — rather than with a graded increase in stenosis severity — and whether combining FAI with circulating inflammatory and metabolic markers improves discrimination beyond either modality alone. Throughout the manuscript the term “anatomically significant stenosis” is used exclusively to denote ≥50% luminal narrowing on CCTA; it does not imply invasively confirmed ischaemia.

## 2 Materials and methods

### 2.1 Ethical approval and patient consent

The study protocol was approved by the Institutional Review Board and Ethics Committee of The First People's Hospital of Kashi Prefecture (Ethics Approval No.: Kashi2021-032; Corresponding Research Project No.: KDYY202413). All procedures complied with the Declaration of Helsinki (2013 revision). All patients provided written informed consent for the clinically indicated CCTA examination and for venipuncture as part of routine care before the procedure. Because the present work is a retrospective analysis of data acquired during routine clinical care and reported in anonymised form, the Ethics Committee granted a waiver of study-specific written informed consent. These two statements are therefore not contradictory: consent covered the clinical procedures, whereas consent specifically for retrospective research use of anonymised data was waived by the Ethics Committee in accordance with national legislation and institutional requirements. The ethics statement in the manuscript front matter has been amended accordingly.

### 2.2 Study population and design

This retrospective observational study included consecutive patients with clinically suspected CAD referred for CCTA at The First People's Hospital of Kashi Prefecture between January 2019 and February 2021. Throughout this manuscript, anatomically significant stenosis refers to anatomically significant luminal narrowing of ≥50% on CCTA and does not imply invasively confirmed ischemia. The primary objective was to examine the relationship between LAD stenosis (prespecified primary outcome: anatomically significant LAD stenosis defined as ≥50% luminal narrowing on CCTA, analysed as a binary variable), lesion-specific perivascular FAI, and systemic inflammatory biomarkers.

The inclusion criteria were: (1) complete baseline clinical and imaging data; (2) LAD stenosis confirmed by CCTA; and (3) no lipid-lowering therapy, including statins, for at least 4 weeks before admission, to reduce treatment-related confounding of inflammatory status.

The exclusion criteria were: (1) prior coronary revascularization or myocardial infarction; (2) cardiomyopathy or severe valvular heart disease; (3) significant stenosis in coronary vessels other than the LAD or coronary anatomical anomalies; (4) suboptimal CCTA image quality precluding reliable interpretation; (5) acute infection, systemic inflammatory disease, or malignancy; and (6) non-statin anti-inflammatory medication use, including systemic corticosteroids, chronic nonsteroidal anti-inflammatory drugs, or colchicine, within 3 months before enrollment. The number of patients excluded at each step of the screening pathway, and the corresponding reason, are given in Figure 1 and in Supplementary Table S0; because the exclusions are applied sequentially, each excluded patient is counted once. Patient selection is summarized in Figure 1.

**Figure 1:**
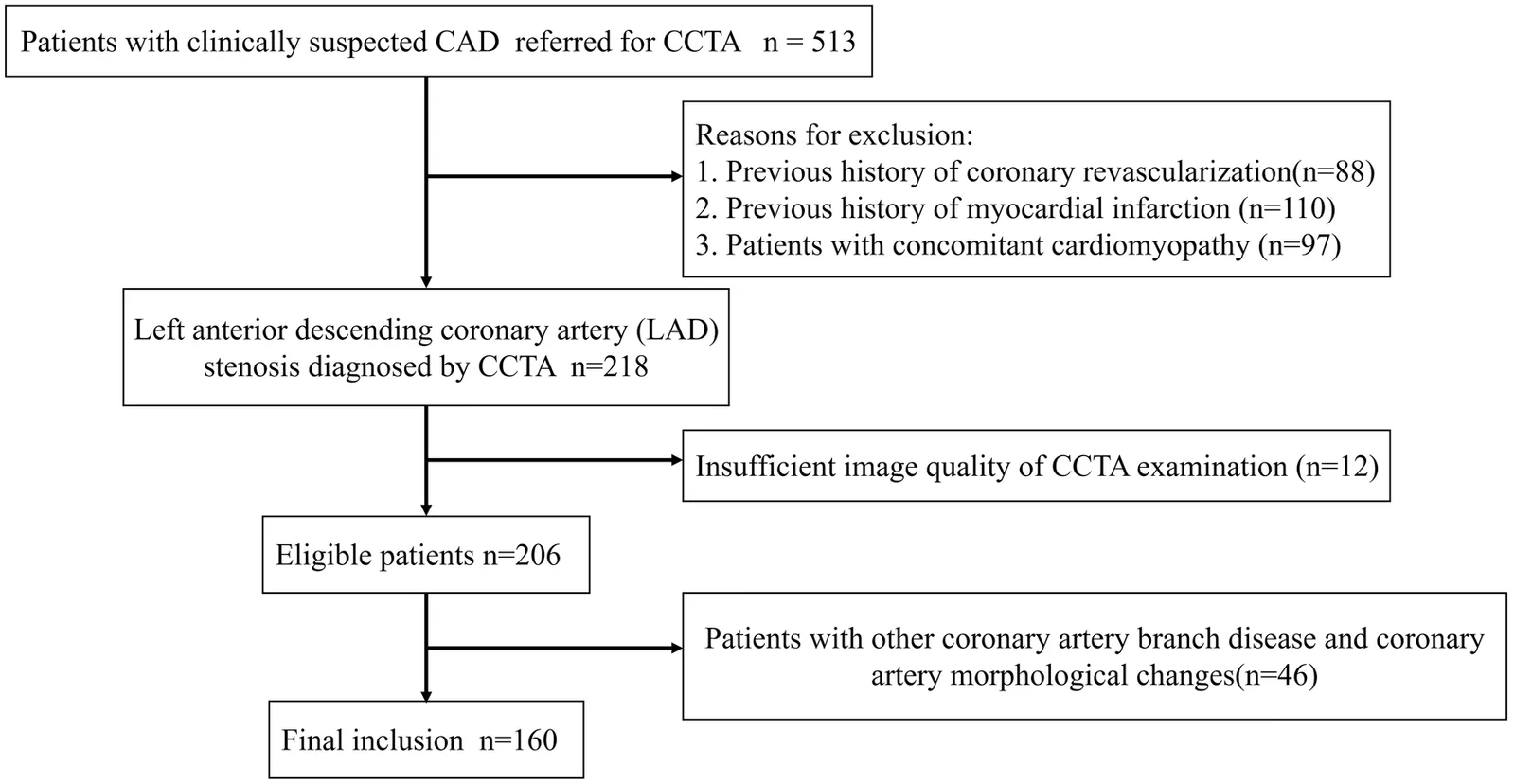
Patient recruitment and selection. From 513 patients with clinically suspected CAD referred for CCTA, 353 (68.8%) were excluded and 160 patients with isolated LAD stenosis met the final eligibility criteria. The number of patients excluded at each step of the screening pathway is given in the flowchart itself and in Supplementary Table S0: 295 patients were excluded before CCTA-confirmed LAD stenosis (previous myocardial infarction *n* = 110, previous coronary revascularization *n* = 88, concomitant cardiomyopathy *n* = 97), 12 for insufficient image quality, and 46 because of significant disease in other coronary branches or coronary morphological changes. The cohort was subsequently divided by stenosis severity: 78 patients with CCTA stenosis <50% and 82 with stenosis ≥50%. CCTA, coronary computed tomography angiography; LAD, left anterior descending artery.

### 2.3 Laboratory measurements

Peripheral venous blood samples were collected within 24 h after CCTA. Laboratory measurements included hs-CRP, LDH, apolipoprotein A1 (ApoA1), apolipoprotein B (ApoB), total cholesterol (TC), and high-density lipoprotein (HDL), all measured according to standardized institutional protocols. The units used throughout this manuscript are: TC, TG, LDL-C and HDL-C in mmol/L; hs-CRP in mg/L; ApoA1 and ApoB in g/L; and LDH in U/L. The unit labels for LDL, HDL and CRP in Table 1 of the previous version of the manuscript were incorrect and have been corrected. The high-sensitivity C-reactive protein assay is referred to as hs-CRP throughout; the abbreviation “CRP” used in the previous version of Tables 1–4 has been replaced by “hs-CRP” for consistency.

**Table 1:** Baseline clinical and biochemical characteristics by stenosis severity.

Variable	Stenosis <50%	Stenosis ≥50%	*χ*Statistic	P
n	78	82		
Ages (years)	59.87 ± 9.82	62.21 ± 11.87	−1.353^b^	0.178
Male	49	60	1.972^a^	0.160
Body mass index	26.43 ± 5.52	25.67 ± 4.66	0.951^b^	0.343
Smoking habit, *n* (%)	20	31	2.551^a^	0.110
blood sugar (mmol/L)	5.57 ± 1.90	6.16 ± 2.91	−1.495^b^	0.137
MAP (mmHg)	94.75 ± 15.15	97.55 ± 13.60	−1.233^b^	0.219
TC (mmol/L)	3.85 ± 1.00	4.19 ± 1.16	−2.004^b^	0.047
TG (mmol/L)	1.79 ± 1.26	1.62 ± 0.88	0.994^b^	0.322
LDL (mmol/L)	2.77 ± 0.85	2.54 ± 1.05	1.501^b^	0.135
HDL (mmol/L)	1.09 ± 0.27	0.99 ± 0.29	2.181^b^	0.031
FAI	−80.10 ± 19.37	−64.58 ± 21.36	−4.807^b^	0.000
hs-CRP (mg/L)	6.87 (5.32,8.37)	15.50 (11.83,19.00)	−8.775^c^	0.000
ApoA1/ApoB	1.26 ± 0.35	0.77 ± 0.36	8.711^b^	0.000
LDH (U/L)	183.03 ± 33.27	241.30 ± 58.66	−7.675^b^	0.000

LDL, low-density lipoprotein cholesterol; HDL, high-density lipoprotein cholesterol; hs-CRP, high-sensitivity C-reactive protein; ApoA1/ApoB, apolipoprotein A1 to apolipoprotein B ratio; LDH, lactate dehydrogenase; FAI, fat attenuation index (HU); MAP, mean arterial pressure.

a chi-square test.

b independent-samples t test.

c Mann–Whitney U test.

**Table 2:** Multivariable logistic regression analysis of stenosis severity.

Variable	B	SE	Wald	OR (95% CI)	P
TC	0.362	0.230	2.478	1.436 (0.915–2.252)	0.115
HDL	−1.290	0.833	2.397	0.275 (0.054–1.409)	0.122
FAI	0.043	0.021	4.421	1.044 (1.003–1.087)	0.035
hs-CRP	0.058	0.029	4.032	1.060 (1.001–1.122)	0.045
ApoA1/ApoB	−1.605	0.581	7.625	0.201 (0.064–0.628)	0.006
LDH	0.014	0.005	9.361	1.014 (1.005–1.024)	0.002
constant	1.104	2.158	0.262	3.017	0.609
Hosmer-Lemeshow test				χ^2^ = 6.82, df = 8, *p* = 0.559	

B, regression coefficient; Wald, Wald chi-square statistic; OR, odds ratio; CI, confidence interval.

**Table 3:** Final multivariable model, diagnostic performance, and internal validation.

Variable	B	SE	Wald	OR (95% CI)	P	AUC (95% CI)	Cut-off	Sens.(%)	Spec.(%)
FAI	0.054	0.020	7.068	1.055 (1.014–1.098)	0.008	0.869 (0.806–0.931)	−71.01	82.9	92.3
hs-CRP	0.061	0.029	4.546	1.063 (1.005–1.125)	0.033	0.902 (0.845–0.959)	10.45	89.0	92.3
ApoA1/ApoB	−1.568	0.559	7.869	0.209 (0.070–0.624)	0.005	0.838 (0.767–0.908)	0.890	81.7	87.2
LDH	0.014	0.005	9.402	1.014 (1.005–1.023)	0.002	0.803 (0.725–0.881)	223.45	76.8	92.3
constant	1.973	1.892	1.088	7.192	0.297	-	-	-	-
Composite Model	-	-	-	-	-	0.915 (0.865–0.965)	0.784	88.5	91.2
Internal Validation - 5-Fold CV	-	-	-	-	-	0.872 (Mean AUC)	-	-	-
- 10-Fold CV	-	-	-	-	-	0.881 (0.826–0.936)	-	-	-
- Bootstrap (1,000×)	-	-	-	-	-	0.895 (0.848–0.942)	-	-	-

The Composite Model incorporates FAI, hs-CRP, ApoA1/ApoB, and LDH; CV = cross-validation; Sens. = sensitivity; Spec. = specificity.

**Table 4:** ROC analysis of individual biomarkers and the composite model.

Variable	AUC	SE	P	95% CI	Cut-off	Sensitivity	Specificity
FAI	0.869	0.032	<0.001	0.806–0.931	−71.01	0.829	0.923
hs-CRP	0.902	0.029	<0.001	0.845–0.959	10.450	0.890	0.923
ApoA1/ApoB	0.838	0.036	<0.001	0.767–0.908	0.890	0.817	0.872
LDH	0.803	0.040	<0.001	0.725–0.881	223.450	0.768	0.923
Composite model	0.915	0.026	<0.001	0.865–0.965	0.784	0.885	0.912

### 2.4 Image acquisition and reconstruction protocol

CCTA was performed using a dual-source CT scanner (Somatom Definition, Siemens Healthineers, Forchheim, Germany). Patients underwent breath-holding training before scanning. Sublingual nitroglycerin was routinely administered for coronary vasodilation, and beta-blockers were used for heart-rate control when resting heart rate exceeded 70 beats/min unless contraindicated. Images were acquired from the tracheal bifurcation to the cardiac base using 120 kVp, automated tube-current modulation (CARE Dose 4D), detector collimation of 2 × 32 × 0.6 mm with Z-Sharp technology, and 0.33-s gantry rotation. Iopromide (Ultravist, 370 mgI/mL; Bayer Healthcare) was injected at 5.0 mL/s followed by a 40-mL saline flush. Images were reconstructed at 0.75-mm slice thickness and 0.5-mm increment using a B26f kernel; multiplanar, curved planar, and volume-rendered reconstructions were generated on a dedicated workstation.

### 2.5 Quantification of perivascular fat attenuation index (FAI)

Lesion-specific perivascular FAI was quantified using an artificial intelligence-assisted post-processing platform (CoronaryDoc, Shukun Technology, Beijing, China). The software generated a three-dimensional volume of interest around the target LAD segment and identified perivascular adipose tissue within −190 HU to −30 HU. The sampling radius extended from the external vessel wall to a distance equal to the local vessel diameter, consistent with CRISP-CT methodology (3, 17). Two experienced cardiovascular radiologists, blinded to clinical and biomarker data, reviewed all segmentations, excluded nonadipose structures, and resolved discrepancies by consensus. Interobserver reproducibility was assessed in a randomly selected subgroup of 40 scans (25% of the cohort) that were independently segmented by both readers; the full results are given in Supplementary Table S5. For the remaining scans a single reader performed the segmentation and the second reader reviewed the output; the mean of the two readers’ values was used as the analytic value in the subgroup with dual readings.

**Definition and localisation of the measured LAD segment.** To make the measurement reproducible, the analysed segment was defined *a priori* and applied identically in every patient:

- **Target lesion.** The target lesion was the LAD segment containing the maximum luminal narrowing identified on CCTA. When more than one LAD lesion was present, the lesion with the greatest percentage diameter stenosis was selected.
- **Segment length and position.** A standardised 40-mm-long segment of the LAD was analysed, centred on the point of maximal luminal narrowing of the target lesion (i.e., extending 20 mm proximal and 20 mm distal to that point). This 40-mm length was chosen to match the proximal-LAD sampling convention used in CRISP-CT (3, 17).
- **Anatomical handling of boundary cases.** When the point of maximal narrowing lay within 20 mm of the LAD ostium, the segment was truncated at the ostium (with a 1-mm offset from the aortic wall to avoid aortic wall partial-volume effects) and extended distally so that the analysed length remained 40 mm. When the lesion lay in the mid-LAD, the same 40-mm rule was applied without truncation. In this cohort the target lesion was located in the proximal LAD in 94 patients (58.8%) and in the mid-LAD in 66 patients (41.2%).
- **Proximal reference.** In patients without a discrete stenotic lesion, the segment was defined as the 40 mm of LAD beginning 1 mm distal to the ostium, so that a standardised region was analysed in every patient irrespective of lesion location.
- **Structures excluded.** The aortic root, the left atrial appendage, the pulmonary trunk, epicardial fat contiguous with the myocardium, and any coronary stent or calcified plaque were manually excluded from the volume of interest. Calcified plaque and contrast within the lumen were excluded by the −190 to −30 HU fat window.
- **Blinding.** Measurements were performed before, and readers were blinded to, the serum biomarker results; readers were also blinded to the clinical indication and to the patient's group assignment.

Because attenuation values are negative, less negative FAI values represent higher perivascular inflammatory burden. Figure 2 illustrates the measurement approach.

**Figure 2:**
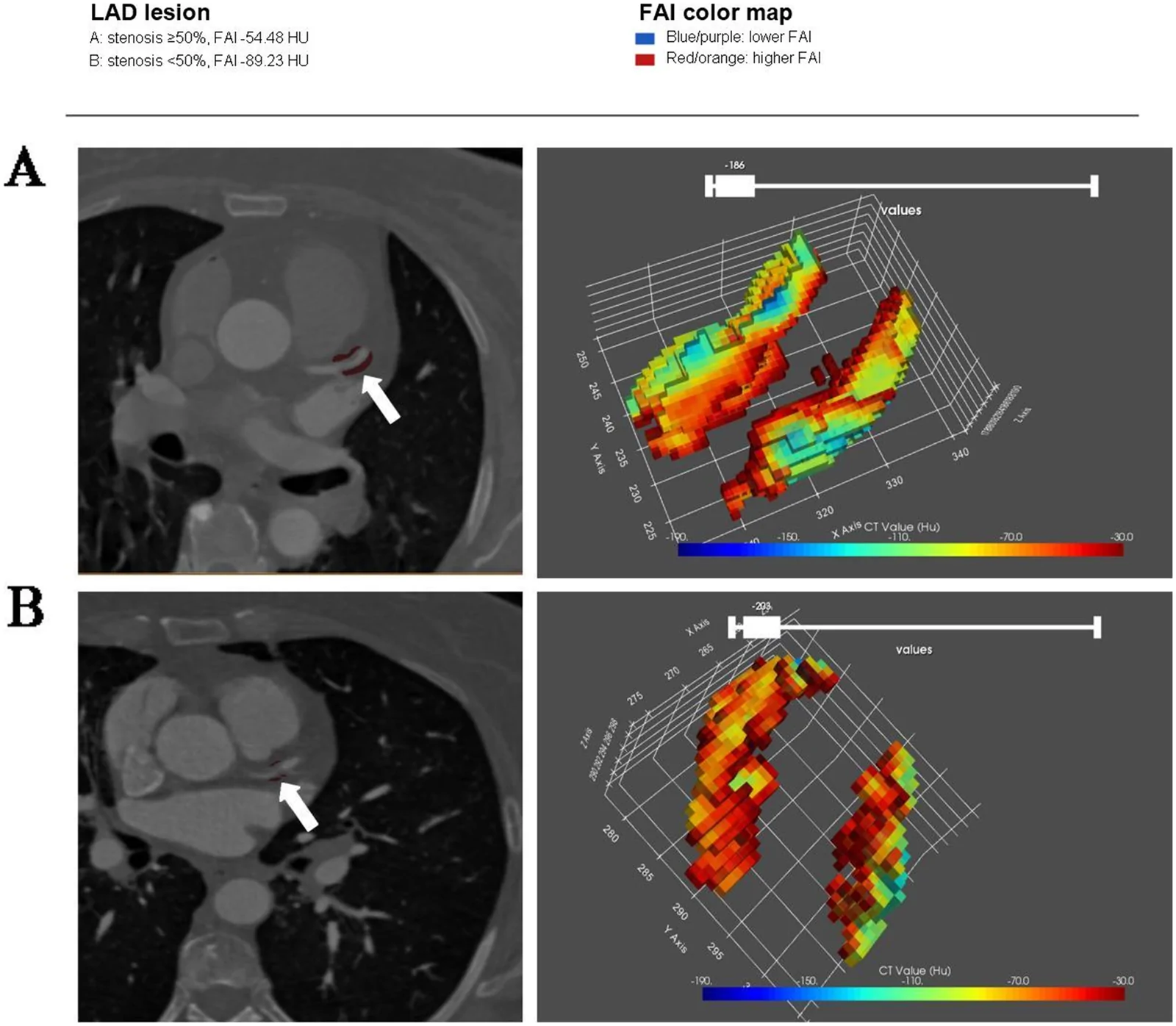
Representative lesion-specific FAI measurements in the LAD. Panel A shows CCTA-defined stenosis ≥50% with higher, less negative perivascular attenuation (FAI, −54.48 HU), displayed predominantly in red. Panel B shows stenosis <50% with lower, more negative attenuation (FAI, −89.23 HU), displayed predominantly in yellow/purple. LAD, left anterior descending artery; FAI, fat attenuation index.

### 2.6 Assessment of CCTA stenosis

#### 2.6.1 Who graded stenosis, and blinding

CCTA stenosis severity was graded by the reporting cardiovascular radiologist for each examination — one of two readers with dedicated cardiovascular CT experience (readers R1 and R2) — working on the dedicated post-processing workstation. Each examination was graded by a single reader, and the same prespecified rule was applied to every patient. Grading was performed as part of routine clinical reporting, before and without knowledge of the serum biomarker results; conversely, the FAI measurements were performed with the reader blinded to hs-CRP, LDH and the ApoA1/ApoB results, so that neither assessment was informed by the other.

#### 2.6.2 How stenosis was determined

Stenosis severity was expressed as the percentage diameter reduction relative to the mean of the proximal and distal reference segments judged to be free of disease, using the standard 18-segment Society of Cardiovascular Computed Tomography model, and was recorded in 1% increments on multiplanar and curved planar reformations. Anatomically significant stenosis was prespecified as ≥50% diameter reduction, applied uniformly to all patients.

#### 2.6.3 Reproducibility

Because each examination was graded by a single reader, no paired independent double-reading record exists from which an interobserver agreement statistic (such as Cohen's *κ*) for the stenosis classification could be derived retrospectively. Rather than report a value we cannot verify, we state this explicitly and have added it as a limitation (Section 4.1). Reproducibility was instead assessed formally for the FAI measurement — the novel and reader-dependent component of this study — in a dual-read subgroup of 40 scans, with results reported in Section 2.5 and Supplementary Table S5 (ICC(2,1) = 0.991, 95% CI 0.986–0.994; Bland–Altman 95% limits of agreement −5.66 to 6.46 HU).

#### 2.6.4 Stenosis as a continuous variable

Because the primary outcome was dichotomised at 50%, the raw percentage stenosis values were also retained and used in the sensitivity analyses described in Section 2.7.

### 2.7 Statistical analysis

Statistical analyses were performed using SPSS version 26.0 (IBM Corp., Armonk, NY, USA), and figures were generated using GraphPad Prism version 7.0 (GraphPad Software, San Diego, CA, USA). Additional analyses (DeLong test, bootstrap resampling, calibration metrics and ordinal regression) were performed in R version 4.3.1 (R Foundation for Statistical Computing, Vienna, Austria) using the packages pROC, rms and rmsb. Continuous variables were assessed for normality with the Shapiro–Wilk test and are reported as mean ± standard deviation or median (interquartile range), as appropriate. Categorical variables are reported as counts and percentages. Between-group comparisons used the independent-samples t test, Mann–Whitney U test, chi-square test, or Fisher exact test, as appropriate.

#### 2.7.1 Primary analysis

The primary outcome was anatomically significant LAD stenosis (≥50% on CCTA) analysed as a binary variable. Spearman rank correlation assessed the association between LAD FAI and stenosis. Multivariable binary logistic regression identified independent predictors of CCTA-defined anatomically significant LAD stenosis. Adjusted odds ratios (ORs) with 95% confidence intervals (CIs) were reported.

#### 2.7.2 Rationale for candidate variable selection

The candidate predictor set was prespecified on biological grounds and comprised the imaging biomarker of interest (FAI) together with the circulating markers that were available in all patients and that have an established or hypothesised link to atherosclerotic inflammation or oxidative metabolism (hs-CRP, LDH, ApoA1/ApoB) and to lipid burden (TC, HDL). Model size was deliberately restricted because the number of events limited the number of predictors that could be estimated reliably (82 events, i.e., 13.7 events per candidate variable in the six-variable model and 20.5 in the final four-variable model); an a-priori decision was therefore made to keep the model parsimonious. This is now stated explicitly, and the consequence of adding further clinical covariates is examined empirically in the sensitivity analysis described below.

Model fit was evaluated with the Hosmer-Lemeshow test.

Diagnostic performance was assessed with receiver operating characteristic (ROC) analysis. Optimal cutoffs were determined by the maximum Youden index. To reduce optimism and test model stability, the final model was evaluated in the same analytic cohort using stratified 5-fold cross-validation, 10-fold cross-validation, and 1,000-bootstrap resampling for bias-corrected AUC estimation.

#### 2.7.3 Nested internal validation

To avoid the optimism that arises when a model is selected on the whole dataset and then “validated” on the same data, the complete model-building procedure — candidate variable set, multivariable logistic regression and variable retention — was repeated *inside* each training partition and each bootstrap replicate, and the resulting model was applied to the held-out data. We report the apparent AUC, the mean optimism, the optimism-corrected AUC, and the proportion of replicates in which each predictor was retained, as a measure of selection stability.

#### 2.7.4 Calibration

Model calibration was assessed in three complementary ways: (i) the Hosmer-Lemeshow test; (ii) a calibration plot of observed versus predicted risk across tenths of predicted risk; and (iii) the calibration slope, calibration-in-the-large (calibration intercept) and the Brier score, with the scaled Brier score (1−Brier/Brier_null) reported as a measure of overall accuracy.

#### 2.7.5 Comparison of ROC curves

Because the composite and single-marker models were evaluated in the same patients, the AUCs are correlated. Pairwise comparisons were therefore performed with the nonparametric DeLong test for correlated ROC curves (18), and differences in AUC with 95% CIs and *P* values are reported. The composite model was compared with hs-CRP and with FAI, the two individual markers with the highest AUCs.

#### 2.7.6 Sensitivity analyses

(i) *Stenosis as a continuous and ordinal outcome.* To address the fact that the primary analysis dichotomises stenosis, we repeated the analysis with stenosis percentage as a continuous outcome using multivariable linear regression, and with an ordinal stenosis grade (0%–25%, 26%–50%, 51%–70%, 71%–100%) using proportional-odds ordinal logistic regression. (ii) *Addition of clinical covariates.* We refitted the model after adding age, sex, current smoking, hypertension, diabetes and fasting glucose; because this expanded model was sparse (8.2 events per candidate variable), estimates were obtained by Firth penalised maximum likelihood to reduce small-sample bias (19), and the change in the four biomarker estimates was examined together with a likelihood-ratio test.

All statistical tests were two-tailed, and a *P* value < 0.05 was considered statistically significant.

#### 2.7.7 Reporting

This study is reported in accordance with the TRIPOD statement (20); the sample-size considerations for prediction-model development follow Riley et al. (21).

## 3 Results

### 3.1 Baseline clinical and biochemical characteristics

Table 1 summarizes baseline clinical and biochemical characteristics by stenosis severity. Compared with patients with stenosis <50%, those with anatomically significant stenosis (≥50%) had higher TC, hs-CRP, LDH, and perivascular FAI values and lower HDL and ApoA1/ApoB ratios (all *P* < 0.05). FAI values were less negative in the ≥50% group, consistent with greater perivascular inflammatory burden (Figure 3).

**Figure 3:**
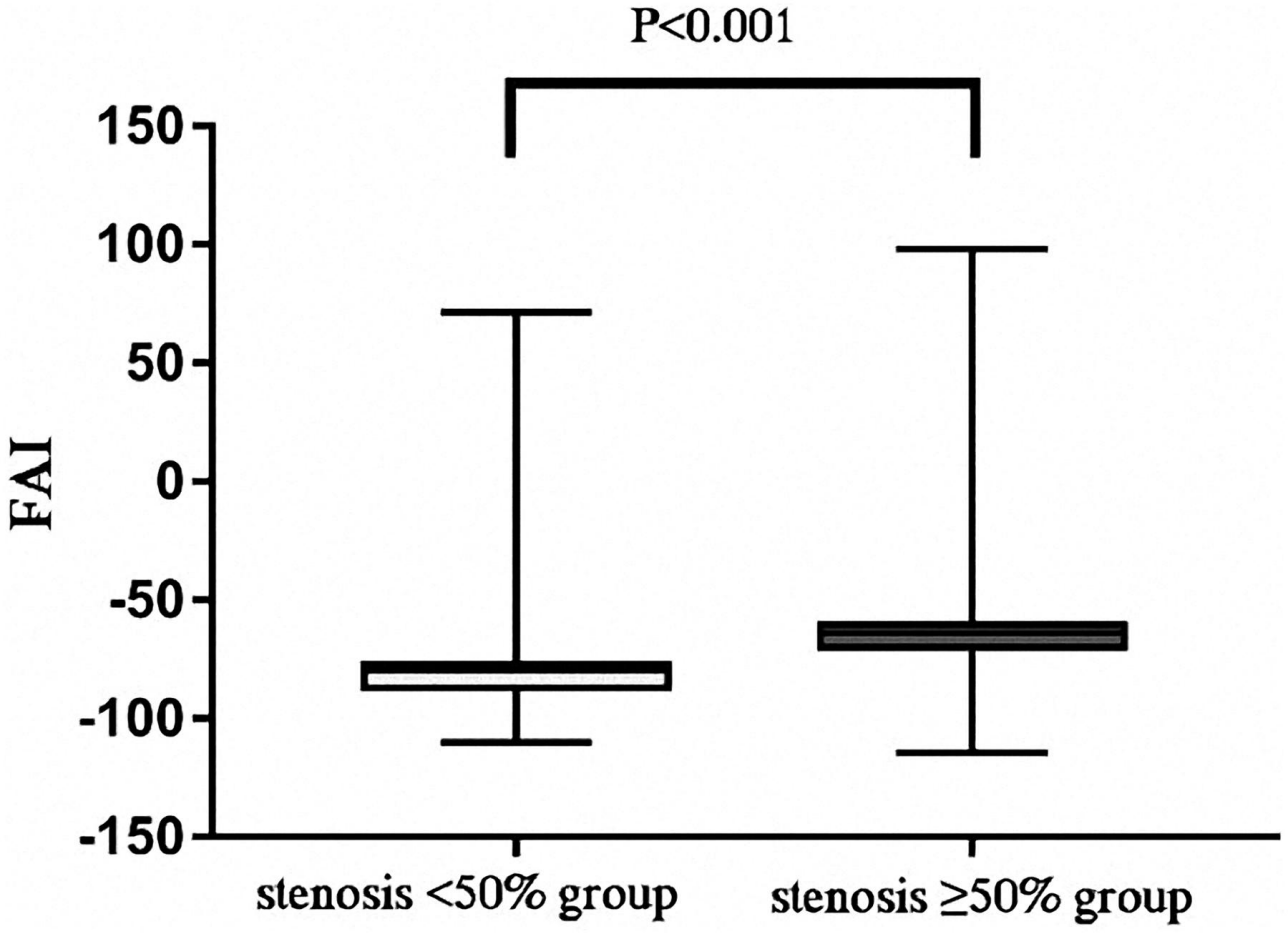
FAI values by stenosis severity. FAI values were significantly higher, that is, less negative, in the stenosis ≥50% group than in the stenosis <50% group (*P* < 0.001).

Using the CRISP-CT cardiac mortality threshold of −70.1 HU as a reference, the mean FAI was higher in the ≥50% group (−64.58 ± 21.36 HU) and lower in the <50% group (−80.10 ± 19.37 HU; *P* < 0.001) (3, 17). This numerical comparison is presented descriptively only: the CRISP-CT threshold was derived for cardiac mortality, in a different cohort, with a different measurement definition and a different endpoint (see Discussion).

### 3.2 Factors associated with stenosis severity

A multivariable logistic regression model including TC, HDL, FAI, hs-CRP, ApoA1/ApoB, and LDH was used to identify factors associated with anatomically significant LAD stenosis. FAI, hs-CRP, and LDH were positively associated with stenosis ≥50%, whereas the ApoA1/ApoB ratio was inversely associated. TC and HDL were not independent predictors. The Hosmer-Lemeshow test indicated acceptable calibration (*χ*^2^ = 6.82, *P* = 0.559).

### 3.3 Development of the composite diagnostic model for CCTA-defined anatomically significant stenosis

To evaluate combined diagnostic performance, a final multivariable model was constructed using the independent predictors identified above. Higher FAI (OR = 1.055, 95% CI: 1.014–1.098), hs-CRP (OR=1.063, 95% CI: 1.005–1.125), and LDH (OR=1.014, 95% CI: 1.005–1.023) were associated with stenosis ≥50%, while the ApoA1/ApoB ratio was inversely associated (OR=0.209, 95% CI: 0.070–0.624). The composite score was Y = 0.054  ×  FAI + 0.061  ×  hs-CRP - 1.568 × (ApoA1/ApoB) + 0.014  ×  LDH + 1.973. Individual AUCs were 0.902 for hs-CRP, 0.869 for FAI, 0.838 for ApoA1/ApoB, and 0.803 for LDH. The composite model achieved an AUC of 0.915 (95% CI: 0.865–0.965), with an optimal cutoff of 0.784, sensitivity of 88.5%, and specificity of 91.2%. Internal validation yielded AUCs of 0.872 with 5-fold cross-validation, 0.881 (95% CI: 0.826–0.936) with 10-fold cross-validation, and 0.895 (95% CI: 0.848–0.942) with 1,000-bootstrap resampling.

#### 3.3.1 Nested internal validation

When the entire model-building procedure — candidate set, multivariable regression and variable retention — was repeated inside each of 1,000 bootstrap replicates and the resulting model was applied to the out-of-bag patients, the mean optimism was 0.019 (2.5th–97.5th percentile of the optimism distribution, −0.010 to +0.057), indicating that the apparent performance of the procedure is only modestly optimistic in this cohort. Selection stability was, however, heterogeneous: hs-CRP was retained in 96.9% of replicates, ApoA1/ApoB in 92.7%, FAI in 81.1% and LDH in only 50.4%. The exact four-predictor set was recovered in 7.9% of replicates and a superset containing all four predictors in 35.6%. The LDH term in particular should therefore be regarded as the least stable component of the model. Full results are given in Supplementary Table S3.

### 3.4 Diagnostic performance of FAI, serum biomarkers, and the composite model

ROC analysis confirmed strong discrimination for individual biomarkers and the integrated model (Table 4, Figure 4). hs-CRP showed the highest single-marker AUC (0.902; 95% CI: 0.845–0.959), followed by FAI (0.869; 95% CI: 0.806–0.931), ApoA1/ApoB (0.838; 95% CI: 0.767–0.908), and LDH (0.803; 95% CI: 0.725–0.881). The composite model provided the highest overall discrimination (AUC, 0.915; 95% CI: 0.865–0.965). This apparent superiority was tested formally and is quantified in Section 3.5.

**Figure 4:**
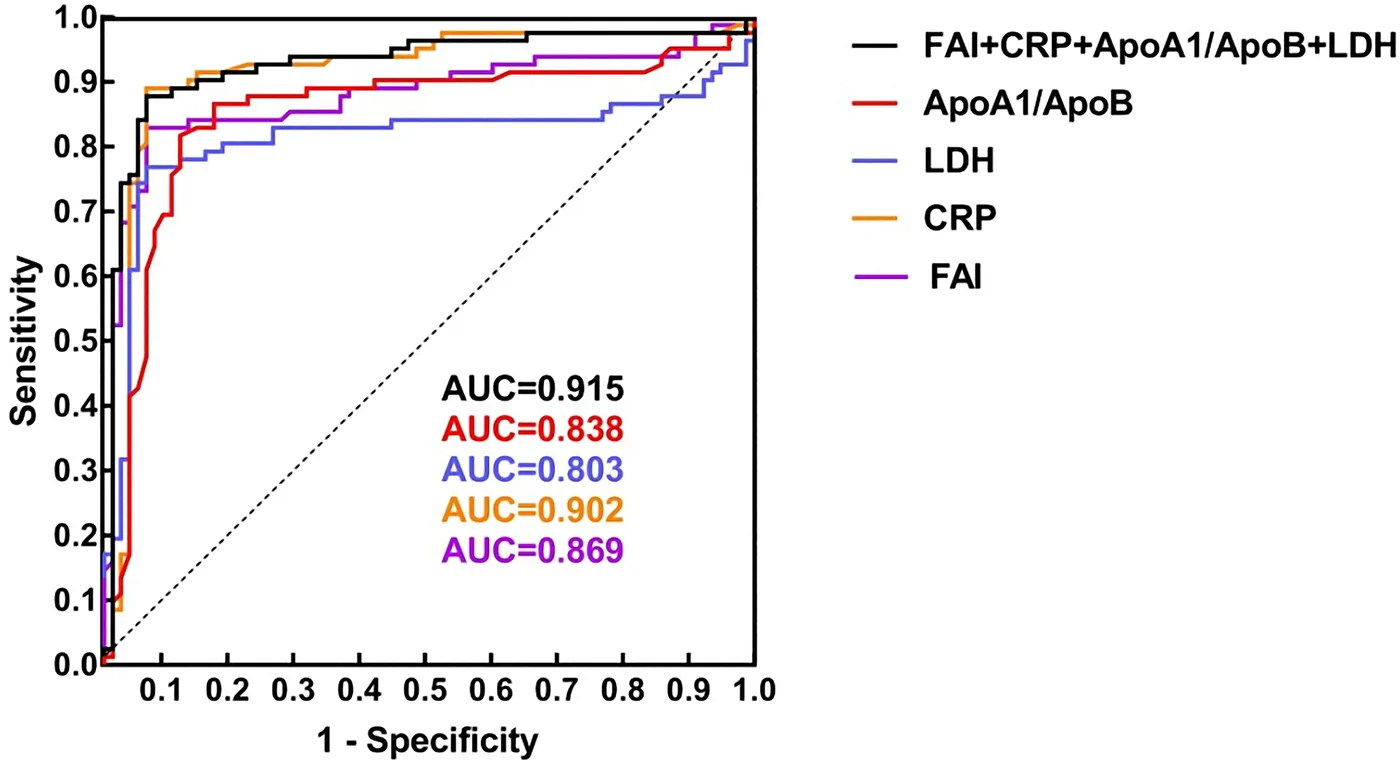
Receiver operating characteristic curves for prediction of CCTA-defined anatomically significant LAD stenosis. In the figure legend the high-sensitivity C-reactive protein curve is labelled “CRP”; throughout the text and tables the same analyte is referred to as hs-CRP.

### 3.5 Formal comparison of ROC curves (DeLong test)

Because all models were evaluated in the same patients, the AUCs are correlated and were compared with the DeLong test (Table 5).

**Table 5:** Pairwise DeLong comparison of correlated ROC curves.

Comparison	z	P
Composite vs. hs-CRP	2.32	0.020
Composite vs. FAI	4.43	<0.001
Composite vs. ApoA1/ApoB	5.00	<0.001
Composite vs. LDH	5.61	<0.001
hs-CRP vs. FAI	3.27	0.001

Each comparison was performed with the nonparametric DeLong test for correlated ROC curves, applied to the model-derived linear predictors in the same 160 patients (18); a positive z statistic indicates that the first-named model had the higher AUC. The AUC corresponding to each model is given in Table 4.

The composite model therefore provides a statistically significant improvement in discrimination over both hs-CRP (the strongest single marker) and FAI. The advantage over hs-CRP is, however, small in absolute terms — the two AUCs are 0.915 and 0.902 respectively — whereas the advantage over FAI is larger. We report both the significance and the magnitude of these differences rather than describing the composite model simply as “outperforming” individual markers.

Multiple pairwise comparisons were performed; because each comparison addresses a distinct, prespecified question (incremental value over the strongest single marker, and over the imaging biomarker of interest), no adjustment for multiplicity was applied, and in the interpretation we rely on the magnitude of the increments as much as on the *P* values.

### 3.6 Model calibration

The Hosmer-Lemeshow test indicated no evidence of gross miscalibration (*χ*^2^ = 8.86, df = 8, *P* = 0.354). Because the Hosmer-Lemeshow test has limited power and is sensitive to the number and choice of groups, calibration was additionally assessed as recommended for prediction models (22). The calibration slope was 1.41 (95% CI 1.00–1.83), that is, above unity. A slope above 1 indicates that the spread of the predicted risks is narrower than that of the observed risks — under-dispersion of the linear predictor rather than overfitting — and implies that the predicted probabilities should not be used as absolute risks in another population without re-estimating the model on local data. Calibration-in-the-large was 0.02 (95% CI −0.46 to 0.50), showing no systematic under- or over-prediction, and the mean predicted probability (0.513) closely matched the observed event rate (0.512). The Brier score was 0.111, with a scaled Brier score of 0.557. Observed and predicted risks agreed closely across all ten deciles of predicted risk (Supplementary Table S4); the calibration plot is shown in Supplementary Figure S1. The mild under-dispersion of the predicted risks, together with the absence of any systematic bias, indicates that the model is reasonably calibrated for this cohort, but re-calibration on local data would be required before use elsewhere.

### 3.7 Sensitivity analyses

**Stenosis as a continuous and ordinal outcome (**Supplementary Table S1**).** When stenosis was modelled as a continuous percentage, FAI remained significantly correlated with stenosis (Spearman *ρ* = 0.54, *P* < 0.001), as did hs-CRP (*ρ* = 0.71, *P* < 0.001) and LDH (*ρ* = 0.49, *P* < 0.001), while the ApoA1/ApoB ratio was inversely correlated (*ρ* = −0.58, *P* < 0.001). In multivariable linear regression with the continuous percentage as the outcome (R^2^  =  0.58), hs-CRP (*β* = 1.48, 95% CI 0.98–1.99, *P* < 0.001), the ApoA1/ApoB ratio (*β* = −20.30, 95% CI −27.14 to −13.46, *P* < 0.001) and LDH (*β* = 0.105, 95% CI 0.051–0.159, *P* < 0.001) remained independently associated, whereas FAI did not (*β* = 0.106, 95% CI −0.020 to 0.233, *P*  =  0.099). A proportional-odds model fitted across four ordinal stenosis grades (0%–25%, 26%–50%, 51%–70%, 71%–100%) gave the same pattern (FAI OR = 1.011, *P*  =  0.16; hs-CRP OR = 1.179, *P* < 0.001; ApoA1/ApoB OR = 0.104, *P* < 0.001; LDH OR = 1.011, *P*  =  0.001). We therefore restrict our claims about FAI to the prespecified binary contrast and do not claim that FAI tracks a graded increase in stenosis severity.

**Addition of clinical covariates (**Supplementary Table S2**).** After adding age, sex, current smoking, hypertension, diabetes and fasting glucose to the final model (Firth penalised likelihood), all four biomarkers retained their direction, magnitude and statistical significance — FAI OR=1.061 (95% CI 1.010–1.114, *P* = 0.018), hs-CRP OR=2.097 (95% CI 1.459–3.015, *P* < 0.001), ApoA1/ApoB OR=0.037 (95% CI 0.005–0.300, *P* = 0.002), LDH OR=1.038 (95% CI 1.010–1.067, *P* = 0.008) — and the FAI estimate changed by less than 2%. None of the six clinical covariates reached statistical significance (age *P* = 0.057, sex *P* = 0.061, smoking *P* = 0.507, hypertension *P* = 0.668, diabetes *P* = 0.501, glucose *P* = 0.685), the global likelihood-ratio test was not significant (*χ*^2^ = 10.70, df = 6, *P* = 0.098), and AIC favoured the parsimonious model (47.33 vs. 48.63). We therefore retained the four-variable model as the primary model and report the expanded model as a sensitivity analysis.

## 4 Discussion

This study evaluated lesion-specific perivascular FAI in relation to LAD stenosis and developed an internally validated model integrating imaging-derived and serum-based biomarkers. Four findings are central. First, FAI, hs-CRP, LDH, and the ApoA1/ApoB ratio were independently associated with CCTA-defined anatomically significant LAD stenosis. Second, the composite model achieved a higher AUC than any individual marker, and this increment was statistically significant on formal paired testing (*P* = 0.020 versus hs-CRP; *P* < 0.001 versus FAI). Third, the direction of the FAI findings was biologically consistent with CRISP-CT. Fourth, model performance remained stable across cross-validation and bootstrap resampling, suggesting limited optimism within the study cohort.

### 4.1 Interpretation of the primary outcome

The primary analysis dichotomised LAD stenosis at 50%. Our findings therefore support an association between perivascular FAI and the *presence* of anatomically significant LAD stenosis, not with a graded increase in stenosis severity. We have revised the title, objectives and conclusions accordingly. When stenosis was analysed as a continuous percentage in a sensitivity analysis, the univariable association with FAI persisted (*ρ*=0.54) but FAI did not retain independent significance after adjustment for the circulating markers (*P* = 0.099). This is compatible with a threshold-like rather than a dose–response relationship, and may also reflect the fact that CCTA percentage stenosis is compressed at both ends of its range. We regard the continuous analysis as exploratory and have moderated our conclusions to match the design.

FAI should be interpreted as a noninvasive surrogate of coronary vascular inflammation, not simply as another measure of luminal narrowing. Inflammatory signaling from the diseased vessel wall suppresses adipogenesis, promotes lipolysis, and increases water content in adjacent perivascular adipose tissue, thereby increasing CT attenuation (28). Because these attenuation values are negative, higher FAI means less negative attenuation and greater local inflammatory burden. This clarification is important for readers who are less familiar with FAI methodology.

The absolute FAI values observed in this cohort support this interpretation. Mean FAI was above the CRISP-CT risk threshold in patients with stenosis ≥50% and below that threshold in patients with stenosis <50% (3, 17). This comparison should be interpreted cautiously: the CRISP-CT threshold of −70.1 HU was derived for cardiac mortality in a different cohort, with a different measurement definition and a different endpoint, whereas the present cohort is a cross-sectional CCTA series. The −71.01 HU value reported here is an internally derived, exploratory threshold: it was identified by the maximum Youden index in the same cohort in which it was evaluated, and it is therefore likely to be optimistic. Bootstrap resampling indicated that the optimal threshold is poorly determined in a cohort of this size, with the 95% interval spanning more than 40 HU. Neither value should be used clinically until it has been validated externally.

### 4.2 Interpretation of the FAI–stenosis correlation

The group-level finding is clinically straightforward: more severe LAD stenosis was associated with less negative FAI values.

The serum biomarkers added complementary information. hs-CRP remained independently associated with anatomically significant stenosis and showed the strongest discrimination among individual circulating markers, consistent with evidence linking chronic low-grade inflammation to atherosclerotic progression and adverse cardiovascular outcomes (9, 12–14, 29). It should be acknowledged, however, that hs-CRP carried most of the discriminative information in this cohort: the increment contributed by adding FAI, LDH and the ApoA1/ApoB ratio to hs-CRP, although statistically significant, was small in absolute terms (AUC 0.915 versus 0.902; *P* = 0.020). The clinical implication is that a single widely available and inexpensive circulating marker already performs well in this highly selected, high-prevalence cohort, and that the added value of imaging the perivascular fat space must be demonstrated in a population with a more balanced case mix before it can be assumed.

LDH was also independently associated with anatomically significant stenosis in the primary model (OR=1.014, 95% CI 1.005–1.023). This association should be interpreted with caution. LDH is a nonspecific enzyme released by many tissues; this study was cross-sectional, did not measure myocardial ischaemia, and did not include serial sampling, so it cannot establish either the source of the enzyme or any temporal relationship. The mechanisms previously proposed — chronic hypoperfusion-driven anaerobic glycolysis and persistent low-grade cardiomyocyte leakage — remain speculative in this design. Although acute infection, recent strenuous exercise and anti-inflammatory medication use were excluded, subclinical inflammation, hepatic congestion, haemolysis and differences in muscle mass were not measured and could contribute. We have therefore removed mechanistic language from the manuscript and present the LDH finding as an observational association that requires confirmation in prospective cohorts with serial measurements and, ideally, ischaemia-specific comparators.

The ApoA1/ApoB ratio also contributed meaningfully to the model. Its inverse association with stenosis severity suggests that the balance between atherogenic particle burden and antiatherogenic lipoprotein capacity may carry information beyond conventional cholesterol concentrations (10, 11). This may explain why TC and HDL did not remain independent predictors after adjustment, whereas the ApoA1/ApoB ratio did.

Restricting the analysis to the LAD was intended to reduce inter-vessel heterogeneity rather than to imply that LAD biology is universally representative of the entire coronary tree. Prior studies have shown regional variation in perivascular fat characteristics across coronary territories (16, 30). A lesion-specific LAD approach therefore improves internal consistency, but future studies should test whether similar biomarker patterns apply to the right and circumflex coronary arteries.

These findings also support a broader research role for CCTA in precision cardiovascular risk stratification. Conventional CCTA defines anatomic stenosis and plaque morphology, whereas inflammation-sensitive biomarkers such as FAI provide information about active vascular biology. Recent work on advanced CT imaging and CCTA-based inflammatory plaque assessment has emphasized that these biomarkers may help identify vulnerable plaques and refine individualized risk assessment beyond luminal stenosis alone (23, 24). Whether integrating lesion-specific FAI with systemic biomarkers can improve the selection of patients for intensified preventive therapy is a hypothesis that our data cannot test: we had no outcome data, no functional ischaemia assessment, and no external validation. We have therefore reframed this section as a hypothesis to be tested rather than a clinical recommendation.

A key strength of this study is the combined assessment of local inflammatory imaging and systemic biomarkers using a prespecified multivariable framework. The final model showed high discrimination and consistent internal validation results. Nonetheless, the model should be viewed as hypothesis-generating and supportive of risk stratification, not as a replacement for established clinical assessment or invasive physiological testing when clinically indicated.

### 4.3 Limitations

Several limitations should be acknowledged.

*Selection bias and generalisability.* Only 160 of 513 screened patients (31.2%) were analysed, and the exclusions occurred at three successive steps — 295 patients before CCTA-confirmed LAD stenosis (previous myocardial infarction *n* = 110, previous revascularization *n* = 88, concomitant cardiomyopathy *n* = 97), 12 for insufficient image quality, and 46 because of significant disease in other coronary branches or coronary morphological changes (Figure 1, Supplementary Table S0). This design decision improved internal consistency but means the cohort is highly selected: patients with multivessel disease — who carry the highest event risk — are systematically absent, and the prevalence of anatomically significant stenosis in the analysed cohort (51.2%) is consequently much higher than in an unselected CCTA population. Because the performance of a diagnostic model depends on prevalence and case mix, the reported AUC, sensitivity and specificity cannot be extrapolated to a consecutive all-comers CCTA population, and the estimates are likely to be optimistic for lower-prevalence settings. External validation in an unselected, multicentre cohort is required.

This was a retrospective, single-center study with strict selection criteria, which may have introduced selection bias and limited generalizability. Stenosis severity was defined anatomically by CCTA, without invasive confirmation of ischemia by fractional flow reserve or other physiological testing; accordingly, anatomically significant stenosis in this manuscript means CCTA-defined luminal narrowing of ≥50% (25–27).

*Absence of outcome data and external validation.* The study endpoint was an anatomical finding on CCTA, not a clinical event. No follow-up, no major adverse cardiovascular event data and no external validation cohort were available. Internal validation, however rigorous, resamples the same patients and cannot detect centre-specific or scanner-specific bias.

*Sample size and model stability.* With 82 events and up to ten candidate predictors in the sensitivity analysis (8.2 events per variable), the study is below the sample size generally recommended for developing a new prediction model (21). Estimates in the expanded model were correspondingly unstable and required penalisation. The primary four-variable model (20.5 events per variable) is at the lower margin of acceptability.

*Threshold derivation.* The FAI and composite-model cut-offs were derived and evaluated in the same cohort and should be regarded as exploratory (see above).

*Reproducibility of the stenosis grading.* CCTA stenosis was graded by a single reader for each examination, with a prespecified ≥50% threshold applied uniformly (Section 2.6). Because no paired independent second reading was archived, a formal agreement statistic for the stenosis classification (such as Cohen's *κ*) could not be computed retrospectively; reproducibility is therefore reported only for the FAI measurement, the novel and reader-dependent element of this study (31) (ICC(2,1) = 0.991, 95% CI 0.986–0.994; Supplementary Table S5). Because the primary outcome is a dichotomisation at a prespecified 50% threshold rather than a fine-grained continuous measurement, the consequences of reader variability are likely to be smaller than they would be for a continuous endpoint, but we are unable to quantify this. Readers who regard a quantified agreement for stenosis grading as essential should note that this would require an independent re-reading of archived CCTAs.

Larger prospective cohorts are required before this model can be considered for clinical implementation.

## Data Availability

The raw data supporting the conclusions of this article will be made available by the authors, without undue reservation.
