# Supplementary material for: Association between the perivascular fat attenuation index and CCTA-defined anatomically significant left anterior descending coronary artery stenosis: relationship with serum inflammatory biomarkers
Source: Front Cardiovasc Med. 2026 Oct 19;13:1823667. doi: 10.3389/fcvm.2026.1823667 (PMC13724379; doi:10.3389/fcvm.2026.1823667)
Supplement: Supplementary file 1 [file Datasheet1.docx]

# **Supplementary Material**

**Manuscript ID:** 1823667

**Title:** Association Between the Perivascular Fat Attenuation Index and CCTA-Defined Anatomically Significant Left Anterior Descending Coronary Artery Stenosis: Relationship With Serum Inflammatory Biomarkers

This file contains Supplementary Table S0 and Supplementary Tables S1–S5 and Supplementary Figure S1.

## **Table S0. Screening outcomes and reasons for exclusion (n = 513 screened)**

| **Screening step** | **Cumulative n excluded** | **n remaining after this step** |
| --- | --- | --- |
| Patients with clinically suspected CAD referred for CCTA | — | 513 |
| minus previous history of coronary revascularization | 88 | 425 |
| minus previous history of myocardial infarction | 110 | 315 |
| minus concomitant cardiomyopathy | 97 | **218** — patients in whom LAD stenosis was confirmed by CCTA |
| minus insufficient image quality of the CCTA examination | 12 | **206** — eligible patients |
| minus other coronary artery branch disease or coronary morphological changes | 46 | **160** — final study cohort |
| **Total excluded / analysed** | **353** | **160** |

Note: These are the same numbers as those shown in the flowchart in Figure 1, read from top to bottom. Each row subtracts the stated number of patients from the number remaining in the row above, so the two columns are cumulative and internally consistent: 513 - 88 - 110 - 97 = 218 patients with CCTA-confirmed LAD stenosis; 218 - 12 = 206 eligible patients; 206 - 46 = 160 patients finally analysed. Of 513 patients screened, 353 (68.8%) were excluded and 160 were analysed. The largest single reason for exclusion was a previous history of myocardial infarction (n = 110), followed by previous coronary revascularization (n = 88) and concomitant cardiomyopathy (n = 97); a further 12 patients were excluded for insufficient image quality and 46 because of significant disease in other coronary branches or coronary morphological changes. Because the exclusions are applied sequentially in the screening pathway, each patient is counted once.

## **Table S1. Sensitivity analysis with stenosis as a continuous and ordinal outcome**

| **Analysis** | **FAI** | **hs-CRP** | **ApoA1/ApoB** | **LDH** |
| --- | --- | --- | --- | --- |
| Spearman ρ vs continuous stenosis %, n=160 | 0.54 (P<0.001) | 0.71 (P<0.001) | −0.58 (P<0.001) | 0.49 (P<0.001) |
| Multivariable linear regression, β (95% CI) | 0.106 (−0.020 to 0.233), P=0.099 | 1.48 (0.98 to 1.99), P<0.001 | −20.30 (−27.14 to −13.46), P<0.001 | 0.105 (0.051 to 0.159), P<0.001 |
| Ordinal (proportional-odds) OR, 4 grades | 1.011, P=0.162 | 1.179, P<0.001 | 0.104, P<0.001 | 1.011, P=0.001 |

Model R² = 0.58. Ordinal grades: 0–25% (n=49), 26–50% (n=36), 51–70% (n=43), 71–100% (n=32).

## **Table S2. Sensitivity model adding clinical covariates (Firth penalised likelihood, n=160)**

| **Variable** | **OR (95% CI)** | **P** |
| --- | --- | --- |
| FAI, per HU | 1.061 (1.010–1.114) | 0.018 |
| hs-CRP, per mg/L | 2.097 (1.459–3.015) | <0.001 |
| ApoA1/ApoB | 0.037 (0.005–0.300) | 0.002 |
| LDH, per U/L | 1.038 (1.010–1.067) | 0.008 |
| Age, per year | 0.919 (0.842–1.003) | 0.057 |
| Male sex | 8.415 (0.909–77.869) | 0.061 |
| Current smoking | 2.012 (0.255–15.898) | 0.507 |
| Hypertension | 1.486 (0.243–9.098) | 0.668 |
| Diabetes | 1.847 (0.310–11.011) | 0.501 |
| Fasting glucose, per mmol/L | 1.093 (0.710–1.684) | 0.685 |

Likelihood-ratio test versus the four-variable model: χ²=10.70, df=6, P=0.098. AIC: 47.33 (four-variable) vs 48.63 (expanded). Firth penalisation was used because the expanded model contained 8.2 events per candidate variable [30].

## **Table S3. Nested internal validation**

| **Metric** | **Value** |
| --- | --- |
| Model-building steps repeated inside each resample | Yes — candidate set, multivariable logistic regression and variable retention |
| Bootstrap replicates | 1000 |
| Mean optimism | 0.019 (2.5th–97.5th percentile, −0.010 to +0.057) |
| Calibration slope (spread of predicted risk) | 1.41 (95% CI 1.00–1.83) — above 1, i.e. predictions under-dispersed |
| Retained — hs-CRP | 96.9% of replicates |
| Retained — ApoA1/ApoB | 92.7% of replicates |
| Retained — FAI | 81.1% of replicates |
| Retained — LDH | 50.4% of replicates |
| Exact four-predictor set recovered | 7.9% of replicates |
| Superset containing all four predictors | 35.6% of replicates |

Note: In each replicate the candidate variable set, the multivariable logistic regression and the variable-retention step were re-run from scratch on the bootstrap sample, and the resulting model was evaluated in the out-of-bag patients. Retention percentages quantify how often each predictor survived this procedure. LDH, retained in only half of the replicates, should be regarded as the least stable component of the model.

## **Table S4. Calibration of the composite model**

| **Decile of predicted risk** | **n** | **Mean predicted risk** | **Observed event rate** |
| --- | --- | --- | --- |
| 1 | 16 | 0.061 | 0.000 |
| 2 | 16 | 0.110 | 0.000 |
| 3 | 16 | 0.160 | 0.188 |
| 4 | 16 | 0.275 | 0.312 |
| 5 | 16 | 0.450 | 0.438 |
| 6 | 16 | 0.589 | 0.562 |
| 7 | 16 | 0.733 | 0.625 |
| 8 | 16 | 0.839 | 1.000 |
| 9 | 16 | 0.932 | 1.000 |
| 10 | 16 | 0.975 | 1.000 |

Calibration slope 1.41 (95% CI 1.00–1.83); calibration-in-the-large 0.02 (95% CI −0.46 to 0.50); Brier score 0.111; scaled Brier score 0.557; mean predicted risk 0.513 vs observed event rate 0.512; Hosmer-Lemeshow χ²=8.86, df=8, P=0.354.

Note: Calibration was assessed for the composite model on the locked analysis dataset. The calibration slope exceeds 1, indicating that the spread of the predicted risks is narrower than that of the observed risks (under-dispersion of the linear predictor); predicted probabilities should therefore not be used as absolute risks in another population without re-estimating the model on local data.

## **Table S5. Reproducibility of FAI measurement**

| **Metric** | **Value** |
| --- | --- |
| Scans independently assessed by both readers | 40 (25% of cohort) |
| ICC model | Two-way random effects, absolute agreement, single measurement (ICC(2,1)) |
| ICC (95% CI) | 0.991 (0.986–0.994) |
| Average-measurement ICC(2,2) | 0.995 |
| Mean difference (reader 1 − reader 2) | 0.40 HU |
| SD of the differences | 3.09 HU |
| Bland–Altman 95% limits of agreement | −5.66 to 6.46 HU |
| Pearson correlation between readers | 0.991 |

ICC confidence interval obtained by nonparametric bootstrap (8000 resamples). Reporting follows Koo and Li [31].

## **Figure S1. Calibration plot**

Observed versus predicted probability of CCTA-defined anatomically significant LAD stenosis across tenths of predicted risk (n = 16 per decile; underlying values in Table S4). Open markers show the observed event rate within each decile with 95% Wilson confidence intervals; the dashed grey line is the line of perfect calibration; the solid red curve is a flexible calibration curve. The model shows no systematic over- or under-prediction, with slight under-spreading of predicted risk at both extremes, consistent with the calibration slope of 1.41 (95% CI 1.00–1.83). LAD, left anterior descending artery.
